# Differential Effects of Light Spectra on Sleep Architecture and Melatonin in Rats

**DOI:** 10.3390/brainsci15050445

**Published:** 2025-04-25

**Authors:** Yuan-Chun Chiu, Pei-Lu Yi, Fang-Chia Chang

**Affiliations:** 1 Graduate Institute of Veterinary Medicine, School of Veterinary Medicine, National Taiwan University, No. 1, Sec. 4, Roosevelt Road, Taipei 106319, Taiwan; cyc822000@gmail.com; 2 Department of Sport Management, College of Tourism, Leisure and Sports, Aletheia University, 32 Zhen-Li Street, Tamsui Dist., New Taipei City 251306, Taiwan; 3 Graduate Institute of Brain and Mind Sciences, College of Medicine, National Taiwan University, Taipei 100233, Taiwan; 4 Graduate Institute of Acupuncture Science, College of Chinese Medicine, China Medical University, Taichung 404328, Taiwan

**Keywords:** blue light, sleep architecture, melatonin, EEG, rat model

## Abstract

Artificial light exposure, particularly from blue-rich sources, has raised concerns about its impact on sleep and circadian rhythms. While blue light’s effects are well-documented, the comparative impact of longer wavelengths, such as orange light (590–635 nm), remains underexplored. This study investigated the effects of 8 h blue (470–490 nm) and orange light exposures (500 lux) on sleep architecture in the next consecutive three days in Sprague-Dawley rats during the light or dark phase of a 12:12 h light–dark cycle. Sleep–wake states were assessed via electroencephalography (EEG) over 72 h. Blue light during the light period suppressed rapid eye movement (REM) sleep acutely and enhanced non-NREM sleep on Days 2 and 3. Orange light during the light period induced no immediate changes but increased NREM sleep on Day 2 with a biphasic REM response—suppression followed by rebound—persisting into Day 3. Blue light during the dark period increased NREM sleep during exposure, followed by suppression in the subsequent light period, with effects normalizing by Day 2. Blue light exposure suppressed melatonin levels compared to controls. These findings highlight spectral and temporal influences on sleep, with blue light exerting stronger acute effects and orange light eliciting delayed, biphasic responses. The results suggest implications for managing light exposure to mitigate sleep disruptions in modern environments.

## 1. Introduction

The proliferation of artificial lighting, particularly blue light (470–490 nm) from light-emitting diodes (LEDs) in electronic devices, has transformed photic environments, raising concerns about its effects on sleep and circadian rhythms [1,2]. Light serves as the primary zeitgeber, synchronizing the suprachiasmatic nucleus (SCN)—the brain’s circadian pacemaker—to the external day-night cycle via intrinsically photosensitive retinal ganglion cells (ipRGCs), which are most sensitive to blue wavelengths (~480 nm) due to the photopigment melanopsin [3,4]. This pathway modulates melatonin secretion, sleep propensity, and arousal, with blue light potently suppressing melatonin and altering sleep patterns when exposure occurs at inappropriate times, such as during the biological night [5,6]. In humans, evening blue light exposure delays sleep onset, reduces REM sleep, and impairs next-day alertness, reflecting disruptions to both circadian (Process C) and homeostatic (Process S) sleep regulation as outlined in the two-process model [7,8].

While blue light’s effects are well-documented, the comparative impact of longer wavelengths, such as orange light (590–635 nm), remains underexplored. Orange light, with lower ipRGC activation, is presumed less disruptive, yet its influence on sleep architecture and circadian timing is unclear [9]. Timing is also critical: light during the active phase (e.g., dark period in nocturnal rodents) mimics human nighttime exposure, potentially amplifying effects, whereas light during the inactive phase (light period in rats) aligns with daytime conditions, where impacts may be subtler [10]. Rat models, with well-defined NREM and REM sleep stages and circadian melatonin rhythms, provide a controlled system to dissect these dynamics, offering translational insights despite their nocturnality [11].

Previous rodent studies indicate that nocturnal blue light increases NREM sleep acutely, possibly as a stress response or circadian shift, followed by compensatory reductions, with REM suppression less consistent [12,13]. Light-induced sleep fragmentation—shorter bouts and more transitions—has been noted, particularly with blue wavelengths, but the persistence and spectral specificity of these effects are uncertain [14]. Human parallels suggest evening screen use disrupts sleep continuity and melatonin, yet systematic comparisons across spectra and exposure timings in controlled settings are lacking [6,15]. Understanding these interactions is vital as modern environments increasingly expose individuals to diverse light spectra at all hours.

This study aimed to evaluate the differential effects of blue and orange light on rat sleep architecture, focusing on (1) immediate and prolonged changes in NREM and REM sleep following 8 h exposures during the light or dark phase and (2) the persistence of these effects over 72 h. We hypothesized that blue light, due to its ipRGC affinity, would acutely suppress REM and increase NREM sleep, with effects varying by timing, while orange light would induce milder, potentially delayed responses. Using EEG recordings, we tested these predictions in Sprague-Dawley rats under a 12:12 h light–dark cycle, providing a foundation to assess light’s spectral and temporal impact on sleep regulation. These findings could inform strategies to mitigate sleep disturbances in artificial-light-dominated settings.

## 2. Materials and Methods

### 2.1. Animals

Male Sprague-Dawley rats (8–10 weeks old, 250–300 g; BioLASCO, Taipei, Taiwan) were used in this study. Rats were housed individually in polycarbonate cages (577.5 cm^2^ × 39.5 cm [height]) under a controlled 12:12 h light–dark cycle (lights on at zeitgeber time [ZT] 0, 300 lx white light; lights off at ZT12) in a temperature-regulated room (23 ± 1 °C) with 50–60% humidity. Food (standard rodent chow) and water were provided ad libitum. Rats acclimated to these conditions for 7 days prior to any experimental procedures to stabilize their circadian rhythms and minimize stress. All animal handling and experimental protocols complied with the ethical guidelines of the Institutional Animal Care and Use Committee (IACUC) at National Taiwan University (approval number: NTU-104-EL-00102).

### 2.2. Experimental Designs

**Figure 1:**
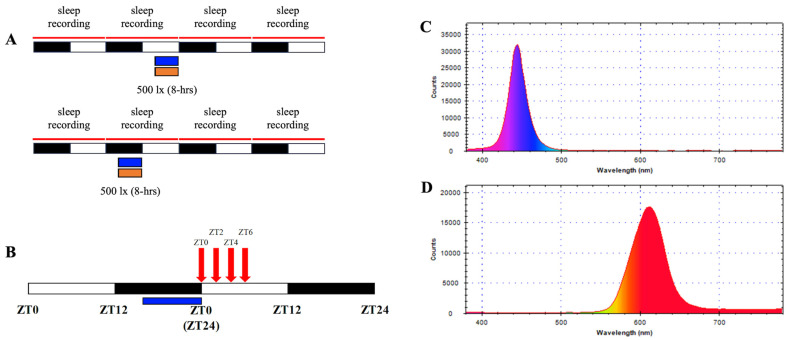
Spectral profiles and experimental design. (**A**) Spectral distribution of blue light (470–490 nm) and (**B**) orange light (590–635 nm) used in the study, measured with a handheld spectrophotometer. Intensity validation showing uniform illumination at 500 lux ± 100 lux across experimental setups. (**C**) Schematic of the experimental protocol, illustrating light exposure timings during the last 8 h of the dark phase (ZT16–ZT24) and the last 8 h of the light phase (ZT4–ZT12). Control groups were exposed to ambient lighting conditions. (**D**) Timeline for melatonin sampling post-blue light exposure, indicating collection points at ZT0, ZT2, ZT4, and ZT6.

Two experiments were conducted:

1. Sleep Behavior Experiment: Twenty-four rats were randomly assigned to blue or orange light groups for light phase (ZT4–ZT12) and dark-phase exposure (ZT16–ZT24) (n = 6 for each group). Another twenty-four rats who received no additional light exposure were used as control to match the light exposure groups (n = 6 for each group). EEG data were collected during 8 h light exposure over 24 h and in the subsequent 24 h, as indicated in Figure 1A.
2. Melatonin Experiment: Six rats received dark-phase blue light, with serum collected at four post-exposure time points. Baseline melatonin data were derived from unexposed controls (n = 5). Protocols are detailed in Figure 1B.

### 2.3. Light Exposure Protocol

Custom-built LED arrays (designed in-house, National Taiwan University) delivered two distinct light spectra: blue (450–490 nm) and orange (590–635 nm). Light intensity was standardized at 500 lx ± 100 lx, measured at the cage floor using a digital luxmeter (LX-1330B, Lutron, Taipei, Taiwan). Spectral profiles were validated with a handheld spectrophotometer (MR-16; TrendForce Corp., Taipei, Taiwan) to ensure wavelength accuracy and minimal overlap between conditions (Figure 1A,B). Each exposure lasted 8 h and was administered in one of two temporal windows: the last 8 h of the dark phase (ZT16–ZT24) or the last 8 h of the light phase (ZT4–ZT12). The experimental designs were shown in Figure 1C. Control groups experienced regular white light (300 lux) without additional light. Exposures occurred in isolated chambers to isolate photic stimuli and eliminate auditory or olfactory confounders. Light sources were mounted 30 cm above the cage floor, ensuring uniform illumination across the experimental space.

### 2.4. EEG Electrode Implantation and Recording

#### 2.4.1. Surgical Procedure

Rats underwent stereotactic surgery to implant electrodes for electroencephalography (EEG) recordings, following established protocols for rodent sleep studies [16]. Surgery was performed under aseptic conditions in a dedicated animal operating suite. Rats were anesthetized with zoletil 50 (100 mg/kg, intraperitoneal (i.p.) injection; Viabac, Carros, France) and xylazine (14.8 mg/kg, i.p. injection; Sigma-Aldrich, Darmstadt, Germany), administered intraperitoneally after confirming loss of the righting reflex. Supplemental doses (20% of the initial dose) were provided as needed to maintain anesthesia, monitored via toe-pinch reflex every 30 min.

The scalp was shaved and disinfected with 75% ethanol and povidone-iodine. A midline incision (2–3 cm) was made from the nasion to the lambda using sterile surgical scissors, and the skin was retracted with micro-clamps to expose the skull. The periosteum was gently scraped away with a sterile cotton swab to reveal bregma and lambda landmarks. The skull was leveled using a stereotaxic frame (Stoelting Co., St. Louis, MO, USA) to ensure precise electrode placement. Three stainless steel screws (1.2 mm diameter, 3 mm length; Plastics One, Torrington, CT, USA) served as EEG electrodes and anchors. Two recording electrodes were positioned over the frontal cortex (anterior–posterior [AP]: +2.0 mm, medial–lateral [ML]: +2.5 mm from bregma) and the parietal cortex (AP: −5.5 mm, ML: +3.0 mm from bregma), primarily targeting frontal areas for optimal delta wave detection during NREM sleep. A reference electrode was placed over the cerebellum (AP: −11.0 mm, ML: −3.0 mm from bregma) to minimize muscle artifacts. Holes were drilled with a micro-drill at low speed to avoid thermal damage, and screws were inserted manually until flush with the dura, ensuring stable contact without penetration.

All electrodes were soldered to a miniature connector (6-pin, Plastics One, Torrington, CT, USA), and the assembly was secured to the skull with dental acrylic cement (Tempron, GC Co., Tokyo, Japan), applied in layers to encase the screws and connector base, leaving the pins exposed for recording. The incision was closed with 4-0 absorbable sutures, and a topical antibiotic (neomycin ointment) and i.p. injection of Penicillin G (20,000 IU/kg, P8721, Sigma-Aldrich) were applied to prevent infection. Rats recovered in individual cages under 12 h:12 h light/dark (L:D) cycle for 7 days, with daily monitoring for weight gain, wound healing, and normal behavior (e.g., grooming, feeding). Rats showing signs of distress or implant failure (e.g., loose connectors, <5% incidence) were excluded and replaced.

#### 2.4.2. Recording and Scoring

After recovery, EEG signals were recorded using a bioamplifier with bandpass filters (V75-01, Coulbourn Instruments, Solana Beach, CA, USA) with 10,000× gain. These filtered signals were transmitted to analog-to-digital conversion (NI PCI-6033E, National Instrument, Austin, TX, USA) at a sampling rate of 128 Hz. EEG signals were filtered (EEG: 0.5–30 Hz) to isolate relevant frequency bands. Recordings began immediately post-exposure and continued for 48 h in an isolated chamber under the standard light–dark cycle. Sleep–wake states were scored manually in 12 s epochs by visual scoring using a custom software (ICELUS V3, M. Opp) written in LabView for Windows (National Instrument) based on standard criteria [16]: wakefulness (mixed EEG frequencies), NREM sleep (EEG is dominant in high-amplitude delta waves, 0.5–4 Hz), and REM sleep (EEG is dominant in theta waves, 6–9 Hz). Scoring was performed blind to experimental conditions by two trained researchers, with inter-rater reliability exceeding 90%.

### 2.5. Melatonin Quantification

Trunk blood samples were collected at ZT0, ZT2, ZT4, and ZT6 post-blue light exposure (n = 5 rats), as shown in Figure 1D. Since our results demonstrated spectral and temporal influences on sleep, with blue light exerting stronger acute effects and orange light eliciting delayed and weak responses, we only determined the alterations of melatonin levels during the acute phase (ZT0-ZT6) after the blue light exposure. Serum was separated by centrifugation (13,000 rpm, 10 min, 4 °C) and stored at −20 °C until analysis. Melatonin concentrations were measured using a commercial rat-specific ELISA kit (ENZ-KIT150, Enzo Biochem, Long Island, NY, USA; sensitivity: 0.162 ng/mL). Samples were thawed and processed per the manufacturer instructions. Absorbance was read at 450 nm using a microplate reader, and concentrations were interpolated from a standard curve (range: 0.1–10 ng/mL). Duplicate measurements ensured precision (CV < 10%).

### 2.6. Statistical Analysis

Sleep percentages (NREM, REM, wake) were expressed as proportions of total recording time and analyzed using one-way analysis of variance (ANOVA) with Duncan’s post hoc test to compare light conditions and controls. Sleep architecture metrics were assessed with paired *t*-tests (exposure vs. baseline within groups). Melatonin concentrations were analyzed via ANOVA with time and condition as factors. Data were processed using SPSS 22.0 (IBM, Armonk, NY, USA), with significance set at *p* < 0.05. Prior to the study, a power analysis was conducted using G*Power 3.1 to estimate the required sample size for detecting medium effect sizes (Cohen’s *f* = 0.25) in sleep architecture variables (NREM, REM, wakefulness) across treatment groups with one-way ANOVA. Assuming an alpha level of 0.05 and power of 0.80, the analysis indicated that a minimum of 6 animals per group would be sufficient to detect statistically significant differences. This estimate accounted for expected within-group variability based on prior studies using similar EEG-based sleep measurements in rodent models.

## 3. Results

### 3.1. Effects of 8 h Blue Light Exposure During the Light Period

**Figure 2:**
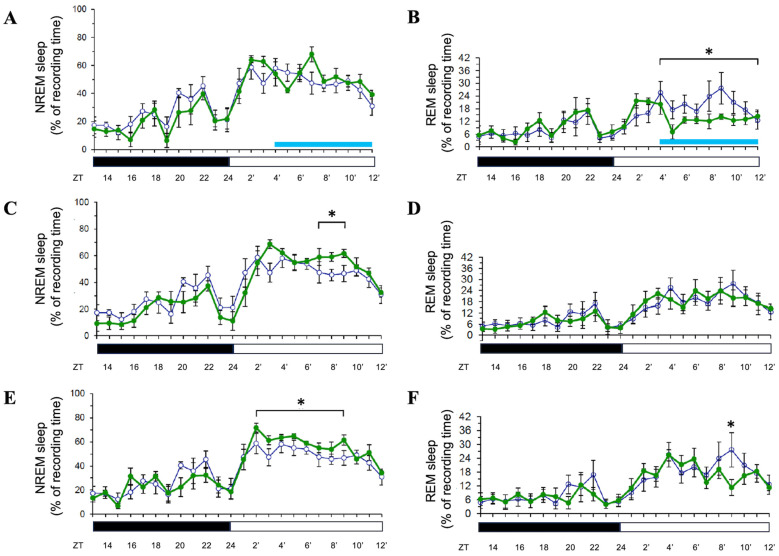
Effects of blue light on NREM and REM sleep during light-phase exposure. The percentage of recording time spent in NREM sleep and REM sleep across zeitgeber times during light-phase exposure to blue light. Data are presented as mean ± SEM for each time point. (**A**,**C**,**E**) represent the percentages of time spent in NREM sleep acquired during the day when receiving blue light exposure (Day 1, Day 2, and Day 3, respectively). (**B**,**D**,**F**) represent the percentages of time spent in REM sleep obtained during the day when receiving blue light exposure (Day 1, Day 2, and Day 3, respectively). White symbols indicate the data collected when rats were exposed to the regular white light (300 lux) in the recording room (the control group), and the green symbols represent the data acquired in the experimental group that received blue light exposure during the particular ZT4-12 on Day 1. Blue bar: the period receiving blue light exposure; black bar: the dark period; white bar: the light period. * indicates *p* < 0.05.

Exposure of Sprague-Dawley rats to 8 h of blue light (500 lux, 470–490 nm) during the light period did not significantly alter non-rapid eye movement (NREM) sleep duration during the exposure itself (Figure 2A). However, rapid eye movement (REM) sleep was significantly suppressed during this period (*p* < 0.05; Figure 2B). On the following day (Day 2), NREM sleep was enhanced during the light period (*p* < 0.05; Figure 2C), while REM sleep showed no significant changes (Figure 2D). This pattern persisted into Day 3, with NREM sleep remaining elevated during the light period (*p* < 0.05; Figure 2E) and REM sleep showing a transient increase only at hour 21 (*p* < 0.05; Figure 2F). These findings indicate a delayed enhancement of NREM sleep post-exposure, with a selective and temporary suppression of REM sleep during the exposure.

### 3.2. Effects of 8 h Orange Light Exposure During the Light Period

**Figure 3:**
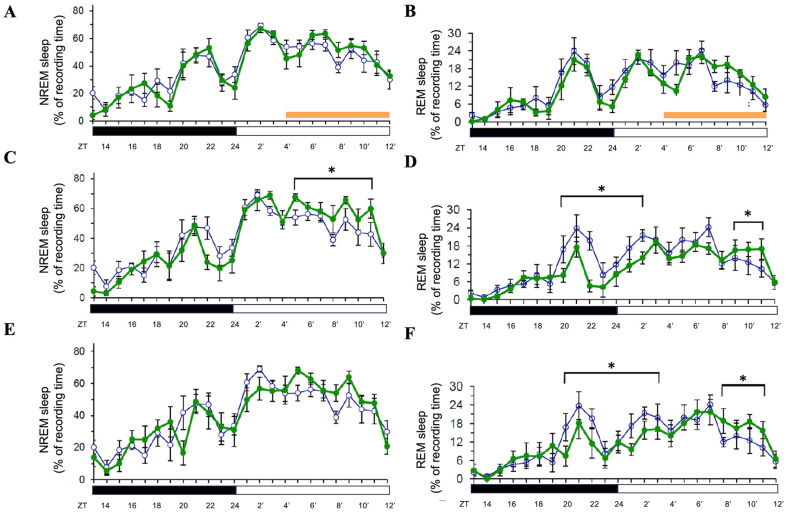
Effects of orange light on NREM and REM sleep during light-phase exposure. The percentage of recording time spent in NREM sleep and REM sleep across zeitgeber times during light-phase exposure to orange light. Data are presented as mean ± SEM for each time point. (**A**,**C**,**E**) represent the percentages of time spent in NREM sleep acquired during the day when receiving orange light exposure (Day 1, Day 2, and Day 3, respectively). (**B**,**D**,**F**) represent the percentages of time spent in REM sleep obtained during the day when receiving orange light exposure (Day 1, Day 2, and Day 3, respectively). White symbols indicate the data when rats were exposed to the regular white light (300 lux) in the recording room, and the green symbols represent the data acquired in the group that received orange light exposure. Orange bar: the period receiving orange light exposure; black bar: the dark period; white bar: the light period. * indicates *p* < 0.05.

In contrast, 8 h orange light exposure (500 lux, 590–635 nm) during the light period did not significantly affect NREM or REM sleep during the exposure (Figure 3A,B). However, on Day 2, NREM sleep was significantly increased during the light period (*p* < 0.05; Figure 3C), mirroring the delayed effect observed with blue light. REM sleep exhibited a distinct pattern: it was suppressed during the last few hours of the dark period and the first few hours of the light period (*p* < 0.05), followed by a rebound increase during the final three hours of the light period (*p* < 0.05; Figure 3D). On Day 3, NREM sleep returned to baseline levels (Figure 3E), while REM sleep followed a similar suppression–rebound pattern as on Day 2 (*p* < 0.05; Figure 3F). These results suggest that orange light induces a delayed NREM sleep increase and a biphasic REM sleep response persisting over multiple days.

### 3.3. Effects of 8 h Blue Light or Ornage Light Exposure During the Dark Period

**Figure 4:**
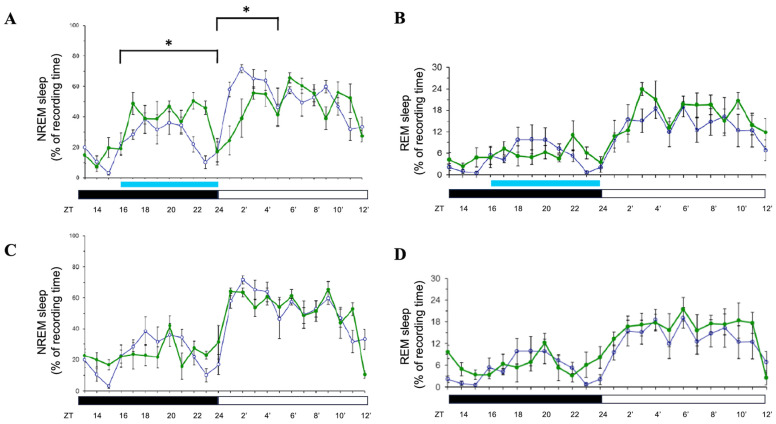
Effects of blue light on NREM and REM sleep during dark-phase exposure. Percentage of recording time spent in NREM sleep and REM sleep across zeitgeber times during dark-phase exposure to blue light. Data are presented as mean ± SEM for each time point. (**A**,**C**) represent the percentages of time spent in NREM sleep acquired during the day when receiving blue light exposure (Day 1 and Day 2, respectively). (**B**,**D**) represent the percentages of time spent in REM sleep obtained during the day when receiving blue light exposure (Day 1 and Day 2, respectively). White symbols indicate the data when rats were exposed to the regular white light (300 lux) in the recording room, and the green symbols represent the data acquired in the group that received blue light exposure. Blue bar: the period receiving blue light exposure; black bar: the dark period; white bar: the light period. * indicates *p* < 0.05.

When rats were exposed to 8 h of blue light (500 lux) during the dark period, NREM sleep significantly increased during the exposure (*p* < 0.05; Figure 4A) but was suppressed during the subsequent light period (*p* < 0.05; Figure 4A). REM sleep remained unaffected during the exposure (Figure 4B). On Days 2 and 3, both NREM and REM sleep returned to baseline levels (Figure 4C,D; data for Day 3 not shown), indicating that the effects of dark-period blue light exposure were transient and confined to the exposure and immediate post-exposure periods. When rats were exposed to 8 h of orange light during the dark phase, both NREM and REM sleep were not significantly altered (personal observation).

Collectively, these findings demonstrate that the timing and spectral quality of light exposure differentially modulate sleep architecture. Blue light during the light period primarily suppresses REM sleep acutely and enhances NREM sleep with a delay, while orange light induces a delayed NREM increase and a biphasic REM response. Blue light during the dark period acutely boosts NREM sleep with a subsequent suppression, without sustained effects. Statistical significance was assessed using paired *t*-tests comparing experimental and control conditions (n = 6; *p* < 0.05).

### 3.4. Melatonin Concentrations Post-Blue Light

**Figure 5:**
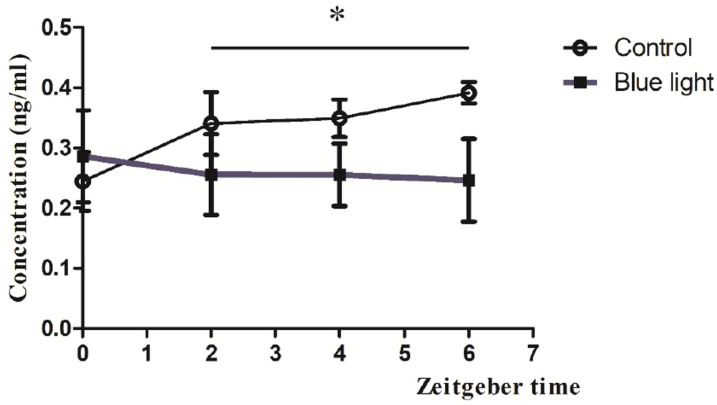
Melatonin suppression by blue light exposure during the dark phase. Serum melatonin concentrations measured at ZT0, ZT2, ZT4, and ZT6 following blue light exposure compared to unexposed controls. Data are presented as mean ± SEM, showing significant suppression at all time points post-exposure. * indicates *p* < 0.05.

Blue light exposure during the dark phase significantly suppressed serum melatonin levels during the subsequent light phase, measured at four zeitgeber time points (ZT0, ZT2, ZT4, ZT6) post-exposure. The concentration of the measured melatonin differed between the control and blue light exposure groups over time. Both groups exhibited similar concentrations at ZT0; the melatonin levels obtained from the control group and blue light exposure group were 0.25 ± 0.02 ng/mL and 0.27 ± 0.02 ng/mL (Figure 5), respectively. However, in the control group, the concentrations of melatonin at ZT2, ZT4, and ZT6 were 0.32 ± 0.05 ng/mL, 0.34 ± 0.04 ng/mL, and 0.38 ± 0.05 ng/mL, respectively. In contrast, the blue light exposure group showed a suppression in melatonin levels; the melatonin concentrations were 0.25 ± 0.04 ng/mL at ZT2 (vs. control at ZT2, *p* < 0.05), 0.24 ± 0.03 ng/mL (vs. control at ZT4, *p* < 0.05), and 0.23 ± 0.05 ng/mL (vs. control at ZT6, *p* < 0.05; Figure 5). Statistical analysis indicated a significant difference between the groups, as denoted by the asterisk in the figure. These findings suggest that exposure to blue light suppresses the melatonin concentration in the subsequent light phase.

## 4. Discussion

This study reveals that 8 h exposures to blue (470–490 nm) and orange (590–635 nm) light at 500 lux differentially affect sleep architecture in Sprague-Dawley rats, with effects varying by timing (light vs. dark period) and spectral quality. These findings reveal the complex interplay between photic stimuli, circadian timing, and sleep regulation, offering insights into how artificial light might influence mammalian physiology.

### 4.1. Blue Light During the Light Period: Selective REM Suppression and Delayed NREM Enhancement

Exposure to blue light during the light period did not alter NREM sleep acutely but significantly suppressed REM sleep, with a delayed NREM increase on Days 2 and 3. The acute REM suppression aligns with prior rodent studies where short-wavelength light reduces REM duration, possibly via ipRGC-mediated SCN activation inhibiting pontine REM generators [1,2]. Given that rats’ inactive phase aligns with light periods, this effect may reflect a mild circadian disruption rather than a robust phase shift, as REM is less sensitive to homeostatic pressure than NREM [3]. The lack of immediate NREM change contrasts with dark-phase findings, suggesting that light-phase exposure—aligned with rats’ natural rest—exerts subtler acute effects.

The delayed NREM enhancement on subsequent days is intriguing, potentially indicating a compensatory homeostatic response or a lingering circadian adjustment. The two-process model posits that Process S (sleep pressure) accumulates during wakefulness and dissipates during sleep, while Process C (circadian timing) modulates sleep propensity [4]. Blue light may have subtly altered Process C, enhancing NREM propensity post-exposure as a rebound effect, though without shifting the overall rhythm, as REM remained largely stable except for a transient increase at hour 21 (ZT9′) on Day 3. This selective impact highlights blue light’s potency, likely tied to ipRGCs’ peak sensitivity (~480 nm) [5], though the delayed nature suggests indirect mechanisms, possibly involving clock gene expression (e.g., *Per2*) or hypothalamic sleep circuits [6].

### 4.2. Orange Light During the Light Period: Biphasic REM and Delayed NREM Effects

Orange light exposure during the light period induced no immediate changes in NREM or REM sleep, but on Day 2, it increased NREM sleep and produced a biphasic REM pattern—suppression early in the light period followed by a rebound, persisting into Day 3. The absence of acute effects contrasts with blue light, reflecting orange light’s lower ipRGC activation due to its longer wavelength (590–635 nm) [5,7]. The delayed NREM increase mirrors blue light’s effect, suggesting a common homeostatic response to light exposure, albeit weaker, possibly driven by cumulative photic stress or subtle SCN modulation [8].

The biphasic REM response—suppression followed by rebound—is notable and may reflect a delayed circadian influence. Orange light, while less effective at suppressing melatonin or activating ipRGCs [7], may still subtly alter SCN timing or arousal systems, reducing REM early post-exposure (e.g., via heightened wakefulness) and triggering a compensatory rebound later. This pattern’s persistence into Day 3 suggests a prolonged but mild disruption, distinct from blue light’s acute REM focus. These findings indicate that even longer wavelengths can influence sleep architecture, challenging assumptions of their neutrality [9].

### 4.3. Blue Light During the Dark Period: Transient NREM Boost and Suppression

Dark-period blue light exposure acutely increased NREM sleep, followed by a suppression in the subsequent light period, with no lasting effects by Days 2 and 3. This acute NREM boost aligns with prior reports of nocturnal light promoting sleep in rodents, interpreted as a stress response or ipRGC-driven phase advance during the active phase [10,11]. Unlike light-period exposure, REM remained unaffected, reinforcing its resilience to immediate photic cues during the dark phase [3]. The subsequent NREM suppression suggests a homeostatic rebound, where elevated sleep during exposure reduced Process S, diminishing sleep pressure post-exposure [4].

The transient nature of these effects—returning to baseline by Day 2—contrasts with light-period findings, indicating that dark-phase disruptions are shorter-lived. This may reflect rats’ stronger circadian entrainment during the active phase, rapidly correcting misalignments [12]. Blue light’s potency here again likely stems from ipRGC activation, though the lack of sustained effects suggests limited circadian phase shifting at this intensity and duration [13].

### 4.4. Mechanistic Insights and Spectral–Timing Interactions

These results suggest two overlapping mechanisms: (1) an acute photic effect, where blue light suppresses REM (light period) or boosts NREM (dark period) via ipRGC-SCN pathways, and (2) a delayed homeostatic or circadian adjustment, driving NREM increases post-exposure across spectra. Blue light’s stronger effects align with its melanopsin affinity [5], while orange light’s milder impact suggests contributions from rods/cones or intensity alone (500 lux) [7,9]. Timing modulates these outcomes: dark-period exposure, misaligned with rats’ activity, elicits immediate NREM changes, while light-period exposure, during rest, yields delayed effects, possibly via subtler SCN or VLPO modulation [14].

The differential REM responses—acute suppression (blue, light period) vs. biphasic shifts (orange, light period)—highlight spectral specificity. Blue light may directly inhibit REM-on neurons [2], while orange light’s delayed pattern could reflect weaker circadian entrainment or secondary arousal effects [8]. The consistent NREM delay across conditions suggests a universal post-exposure adjustment, potentially linked to adenosine buildup or clock gene dynamics [6,15].

### 4.5. Possible Mechanisms of Melatonin Suppression by Blue Light Exposure and Implications for Health and Circadian Regulation

At ZT0, both groups exhibited similar melatonin levels, indicating that baseline concentrations were unaffected by prior exposure. However, the blue light exposure group demonstrated suppressed melatonin levels at all subsequent time points, with statistically significant differences observed. This suppression suggests that blue light exposure disrupts normal circadian signaling and melatonin synthesis [17,18]. The suppression of serum melatonin levels post-blue light exposure can be attributed to the high sensitivity of melanopsin-containing retinal ganglion cells to short-wavelength light. These cells relay photic signals to brain regions regulating circadian rhythms and melatonin production. Blue light exposure during the dark phase likely disrupts this signaling pathway, leading to altered melatonin dynamics during the subsequent light phase [17,19]. Furthermore, studies have shown that even brief exposures to blue light can elicit dose-dependent suppression of melatonin [17], revealing its potency.

The suppression of melatonin has broader implications for health and circadian regulation. Melatonin is critical for maintaining sleep–wake cycles and regulating physiological processes tied to circadian rhythms. Chronic disruption of melatonin secretion due to blue light exposure may contribute to sleep disturbances, impaired alertness, and increased risk for metabolic and cardiovascular diseases [20,21,22]. Moreover, these findings emphasize the importance of minimizing blue light exposure during nighttime or dark phases to preserve natural circadian functioning.

### 4.6. Translational Relevance and Implications for Human Sleep Hygiene

The spectral- and timing-dependent effects of light exposure observed in this study have significant implications for human sleep hygiene, particularly in environments saturated with artificial lighting and screen use. Although conducted in nocturnal rodents, the suppression of REM sleep and alterations in NREM sleep following blue light exposure align with human studies demonstrating that evening exposure to blue-rich light from devices like smartphones and computers can reduce deep sleep and impair sleep quality. For instance, Ishizawa et al. (2021) found that pre-bedtime blue-light exposure decreased the ratio of deep sleep in healthy young men, suggesting that even short-term exposure can have lasting effects on sleep architecture [23].

Moreover, interventions such as blue-light blocking glasses have shown promise in mitigating these effects. Hester et al. (2021) reported that wearing blue-blocking glasses in the evening improved sleep by facilitating the onset of melatonin production, highlighting a practical strategy for reducing the impact of artificial light on circadian rhythms [24].

These findings underscore the importance of managing light exposure in the evening to maintain healthy sleep patterns. Implementing behavioral and environmental strategies, such as using blue-light filters, adopting “night mode” settings on devices, and opting for amber-toned lighting, can serve as non-pharmacological interventions to protect circadian rhythms and enhance sleep quality in humans.

### 4.7. Implications and Limitations

These findings have implications for understanding artificial light’s impact, particularly blue-rich sources prevalent in modern environments. The acute REM suppression and delayed NREM effects of blue light parallel human sleep quality declines post-evening screen use [1,2], despite rats’ nocturnality. Orange light’s subtler effects suggest it as a less-disruptive alternative, though its biphasic REM influence warrants further study. The transient dark-period effects indicate that timing exacerbates acute disruptions, relevant for night-shift or late-night light exposure scenarios.

Limitations include the acute 8 h design, which may not reflect chronic exposure typical of daily device use. The 500 lux intensity exceeds common indoor levels (100–300 lux), potentially amplifying effects. The rat model’s nocturnality limits direct human extrapolation.

### 4.8. Future Directions

Future research should explore chronic exposures and vary intensities to establish dose–response relationships. Measuring melatonin and clock gene expression could confirm circadian involvement, while multi-day recordings might reveal cumulative effects. Extending to diurnal models or humans could bridge translational gaps. Investigating orange light’s REM dynamics and testing dim-light controls would refine spectral specificity.

### 4.9. Conclusions

This study provides critical insights into the differential effects of blue and orange light on sleep architecture and melatonin levels in rats, highlighting the spectral and temporal influences of artificial light exposure. Blue light exposure during the light period acutely suppressed REM sleep and induced a delayed enhancement of NREM sleep, with effects persisting into subsequent days. Conversely, orange light elicited milder, delayed responses, including a biphasic REM sleep pattern and increased NREM sleep on Day 2. Exposure to blue light during the dark period disrupted circadian rhythms by suppressing melatonin levels and altering subsequent sleep patterns, while orange light had less pronounced effects.

These findings underscore the importance of considering both the spectral composition and timing of artificial light exposure in managing sleep disturbances. The results suggest that blue light exerts stronger acute effects on sleep regulation, whereas orange light may offer a less disruptive alternative for nighttime environments. This study offers a foundation for developing evidence-based guidelines to mitigate the adverse impacts of artificial lighting in modern settings.

## Data Availability

Data are available upon request by contacting the corresponding author.

## Abbreviations

AP	anterior–posterior
EEG	electroencephalogram
ip	intraperitoneal
ipRGC	intrinsically photosensitive retrinal ganglion cell
LED	light-emitting diodes
ML	medial–lateral
NREM	non-rapid eye movement
REM	rapid eye movement
SCN	suprachiasmatic nucleus
ZT	zeitgeber time
